# Disadvantaged Parents’ Engagement with a National Secondhand Smoke in the Home Mass Media Campaign: A Qualitative Study

**DOI:** 10.3390/ijerph13090901

**Published:** 2016-09-09

**Authors:** Neneh Rowa-Dewar, Amanda Amos

**Affiliations:** 1 Centre for Population Health Sciences, Usher Institute, University of Edinburgh, Edinburgh EH8 9AG, UK; 2 UK Centre for Tobacco and Alcohol Studies, Centre for Population Health Sciences, Usher Institute, University of Edinburgh, Edinburgh EH8 9AG, UK; amanda.amos@ed.ac.uk

**Keywords:** mass media, secondhand smoke, parents, health inequalities, smoking in the home

## Abstract

Mass media campaigns can be effective in tobacco control but may widen health inequalities if they fail to engage disadvantaged smokers. This qualitative study explored how parents with young children living in disadvantaged circumstances engaged with a national campaign which aimed to raise awareness of the importance of smokefree homes. Individual semi-structured interviews were carried out with 17 parents before and after the Scottish 2014 “Right Outside” mass media campaign. A conceptual framework exploring meaningful exposure (recall and understanding), motivational responses (protecting children from secondhand smoke (SHS)) and opportunities to act (barriers) was used to thematically analyse the findings. Campaign recall and engagement, and motivation to protect children were high. Parents identified with the dramatized scenario and visual impact of SHS harm to children in the TV advertisement. Some reported changed smoking practices. However, supervising young children in limited accommodation when caring alone constrained opportunities to smoke outside. Instead, parents described actions other than smoking outside that they had taken or were planning to take to create smokefree homes. Mass media campaigns using emotive, real-life circumstances can be effective in engaging parents about SHS. However, the behavioural impact may be limited because of difficult home environments and circumstances.

## 1. Introduction

Substantial international evidence on the negative health effects of exposure to secondhand smoke (SHS) exists, particularly for children for whom these effects include reduced lung function, asthma, glue ear, and bronchitis [1,2]. Reducing children’s SHS exposure is, therefore, an important aim of tobacco control. SHS exposure reduced in adults and children following the implementation of comprehensive smokefree public places policies in countries such as Scotland (in 2006) and England (in 2007) [3,4,5]. However, smokefree policies do not encompass private homes where children spend most of their time. Further, the decline in SHS exposure has been greatest in socioeconomically advantaged groups, thereby increasing social inequalities in children’s SHS exposure [5]. In Scotland, 33% of the most socioeconomically disadvantaged children are exposed to SHS in homes compared to 6% of the most advantaged children [6]. This reflects higher parental smoking rates and fewer smoking restrictions in disadvantaged homes [7]. While disadvantaged parents attempt to reduce children’s SHS exposure by restricting smoking to one room and/or to when children are not present [8,9,10,11], only creating and maintaining a smokefree home effectively protects children [12]. However, a recent systematic review of the barriers, motivators, and enablers to smokefree homes concluded that these include: knowledge, awareness and risk perception; agency and personal skills/attributes; wider community norms and personal moral responsibilities; social relationships and influence of others; perceived benefits, preferences and priorities; addiction and habit; and practicalities [13]. While some of these apply to all study settings and populations, the increasing introduction of smokefree legislation has shown that disadvantaged parents can face several barriers to creating smokefree homes, including limited access to safe outside space, more permissive smoking norms and limited awareness/ acceptance of SHS messages [8,9,10,11].

In 2014, Scotland became the first country to set a national target to reduce the number of children exposed to SHS in the home, from 12% to 6% by 2020 [14]. The announcement of this target was supported by the launch of a national mass media campaign “Right Outside” which aimed to increase awareness about the importance of a smokefree home to reduce children’s SHS exposure. Mass media campaigns, when designed appropriately, can be effective as part of tobacco control programmes [15]. For example, smoking cessation mass media campaigns have increased awareness of the harms of smoking, changed smoking attitudes and norms, increased quitting intentions and quit attempts, and reduced adult smoking prevalence [16,17]. SHS mass media campaigns have not been evaluated as extensively or as comprehensively as those on cessation [18]. However, an international review of more than 30 SHS mass media campaigns suggested that the more successful campaigns used television, relied on “testimonials” or personal stories, focused on the health impact of SHS on children, and elicited negative emotions while not demeaning smokers [18], and a recent UK study indicated a tentative and temporary effect of secondhand smoke campaigns on smokefree homes [19]. TV advertisements containing graphic health harm messages were also found to be the most effective in increasing motivation for creating smokefree homes in male smokers and non-smokers in China, India, and Russia [20].

Concerns have been raised that mass media campaigns may inadvertently increase health disparities due to an unequal uptake of the messages by different socioeconomic status (SES) groups, and inequalities in social resources to support quitting smoking [21,22] or the ability to smoke outside the home [11]. While evidence on the equity impact of SHS mass media campaigns is limited, and a recent large UK survey study found no evidence of participants’ socioeconomic status affecting the likelihood of them creating a smokefree home after secondhand smoke mass media campaigns [19], a review of cessation campaigns found that disadvantaged populations were more likely to respond to negative imagery or testimonials about the negative effects of smoking [23]. Advertisements which stir emotional reflections about one’s own behaviour or life may be particularly persuasive, but the emotional reaction has also been found to depend on the extent to which individuals relate to the characters and/or story portrayed in both cessation [24] and SHS campaigns [18].

The impact of a mass media campaign is usually measured by levels of recall and/or changes in awareness, attitudes, or behaviour [21]. Niederdeppe and colleagues [21] have identified three different areas of relevance when exploring the effect in different SES groups: meaningful exposure, motivational response to messages, and the opportunity to act on them. Meaningful exposure relates to the comprehension and recall of the message(s). Smokers in disadvantaged circumstances may have lower exposure as they may have less access to media (e.g., Internet), or use or attend to media differently. The motivational response to the message(s) relates to whether the campaign messages increase motivation to seek further information, advice and/or support to implement the message by, for example, phoning quit lines or exploring websites. Opportunities to act acknowledges that people from different SES groups may be constrained in responding to the message(s) communicated, as lower SES groups have fewer social (e.g., social capital, networks, workplace cultures) and structural (e.g., healthcare access) resources to support behaviour change even when motivation to change is high [25]. This analytical framework is particularly relevant to assess disadvantaged parents’ responses to a smokefree homes campaign, as previous research has shown that opportunities to act might be constrained by their limited domestic and social circumstances [26,27]. Concerns have also been expressed about the unintended consequences that tobacco control interventions, including mass media, can have such as increasing guilt and stigma, particularly among disadvantaged mothers [28,29]. Disadvantaged parents of young children thus represent a target group for smokefree mass media campaigns, but may experience particular barriers in responding to the messages. However, as far as we are aware, no studies have used qualitative methods to explore how parents who live in disadvantaged circumstances engage with and respond to mass media campaign messages on SHS in the home. This is surprising as effective targeting of specific populations requires an in-depth understanding of current awareness, reasoning around current protective measures and barriers to responding to smokefree home messages [18].

The Scottish Government’s “Right Outside” media campaign aimed to increase awareness of SHS and the importance of smokefree homes to protect child health. The key messages were children’s greater vulnerability to SHS and the ineffectiveness of many parents’ existing attempts to protect children by restricting smoking to one room and/or dispersing the smoke through an open window. The campaign had several elements including community events, radio advertisements, posters and a website (http://www.rightoutside.org). The main element of the campaign was a 40-s TV advertisement which aired up to 10 times a day from 26 March to 15 June 2014 on three terrestrial TV channels. The advert showed a mother tucking her six-year old son into bed, shutting his bedroom door and the kitchen door before lighting a cigarette. She opens the window and when her partner enters she gestures to him to shut the door quickly, and waves her hand to disperse the smoke. She then puts the cigarette out and closes the window. A split screen shows on one side her watching TV with her partner, brushing her teeth and going to bed, while on the other side tobacco smoke gradually darkens her son’s lungs as shown on his white t-shirt while he sleeps. A voice-over explains that *“Kids breathe faster than adults. When you smoke indoors, your secondhand smoke lingers in the air. You can’t see it or smell it but it’s there. No matter what you do the harmful chemicals move from room to room for up to five hours, and because your child breathes faster than you, they breathe more of those harmful chemicals. You can choose whether your child breathes secondhand smoke or clean air. For your kids’ sake, don’t smoke indoors. Take it right outside”.*

In this study we first undertook interviews with disadvantaged parents prior to the media campaign and found that the challenging and changing living circumstances and the increasing mobility of children in their first few years were key barriers to them creating smokefree homes [30]. Our previously published paper on these findings focussed on the disadvantaged mothers who were interviewed and their reported attempts to protect their children from both SHS and becoming smokers. We found that these attempts were motivated by the perceived future health and financial burdens these entail [30]. In the context of several intersecting dimensions of disadvantage (unemployment, low income, alcohol/drug abuse, and domestic abuse), the imperative to be and to be seen to be a good mother was also key in shaping smoking practices in the home. While some recognised that the strategies that they used to protect their children from SHS (e.g., opening windows, moving to another room) were not wholly effective, they were perceived to be the best that they could do within their constrained circumstances. In this paper we draw on the second wave of interviews with a sub-sample of these parents (including fathers) which took place after the media campaign and explore how they engaged with, and responded to, this mass media campaign which encouraged them to smoke “right outside”, given their challenging circumstances, drawing on an adapted version of Niederdeppe et al.’s framework [21] outlined previously.

## 2. Materials and Methods

### 2.1. Participants

Twenty-two mothers and three fathers were interviewed individually pre-campaign in November 2013–January 2014. The post-campaign follow-up interviews with seventeen of these parents took place in May–August 2014. Eight of the original parents could not be contacted or did not turn up for the interview. Fourteen mothers and three fathers aged 22–47 years were interviewed. Parents had one to four children of whom at least one was under three years old. Fourteen were single parents, all were long-term unemployed, and all but one parent lived in a flat without direct access to outside space. All parents smoked apart from four mothers who had quit smoking a few weeks prior to the first interview, two of whom had relapsed by the second interview.

The parents were purposively recruited in the first pre-campaign phase of the study from Early Years’ Centres in five Edinburgh communities; three disadvantaged and two of mixed SES. Centres were contacted by the interviewer (NR-D) to inform staff about the study. Parents who smoked and had children aged 1–3 years were informed by their key worker first, and then by the interviewer, that their voluntary participation would entail an interview at a place and time of their choice about where, when, and why they smoked. Parents are referred to centres by social services when considered vulnerable and in need of support, usually because of issues related to mental health, drug or alcohol dependency, and/or family breakdown. Participants were informed verbally and in writing in the first phase of the study, that the interview would explore smoking practices around young children in the home. In the second phase, participants re-consented to be interviewed about any changes in their smoking practices and their views on anything they had seen on SHS in the media. Participants were advised before both interviews that they could withdraw from the study at any time and decline to answer any question. Written consent was obtained. Ethical approval was granted by the University of Edinburgh Centre for Population Health Ethics Committee. Participants received a £15 voucher per interview to recognise their participation in the study. Participants’ names have been changed to pseudonyms to protect their anonymity.

### 2.2. Data Collection Methods

Interviews took place in private rooms in the centres, lasted 25–75 min (average length 45 min), and were digitally recorded with participants’ permission. In the first interview, parents were asked about their understandings of SHS, smoking restrictions in their home, interactions with others around these, and any changes over time. Accounts of home smoking restrictions were prompted using floor plans, where parents drew a floor plan of their home and indicated where they smoked during pregnancy, after their child was born, and in the period since then. This method was developed in a previous smoking in the home study [10].

In the follow-up interviews, which are the focus of this paper, the interview guide (see supplementary materials) covered questions regarding any changes since the first interview to parents’ smoking practices and understandings about SHS. If home smoking restrictions had changed they were asked to illustrate the change(s) in another floor plan. They were then asked if they had heard or seen anything about secondhand smoking or smoking in the home since the first interview and, if so, what they remembered. Participants who had seen the TV advert were asked their views about the messages, and if and how they could implement them. At the end of the interview, after being asked about their recall and the impact of the advert, all participants were shown the TV advert on an iPad. Those who had previously seen it were asked about any additional responses to it, and those who had not seen it were asked about their responses to it.

### 2.3. Data Analysis

The analysis focused on participants’ perspectives on the campaign in the second post-campaign interviews. The digital interview recordings were transcribed, and the transcripts read and reread by both authors alongside the field notes of the first author who conducted the interviews. Emerging themes and issues were identified by the first author according to Braun and Clarke’s approach to thematic analysis, a flexible and iterative method for identifying, analysing, and reporting patterns as themes within qualitative data [31]. The themes were verified by the second author and the final comparative analysis involved both authors. After a full thematic analysis of participants’ accounts, the authors analysed participants’ accounts of their responses to the mass media campaign using Niederdeppe et al.’s [21] framework of meaningful exposure, motivational response, and opportunity to act. We have adapted the motivational response category of the framework to also encompass increased changes in awareness and/or smoking practices. The quotations from the interviews included in the results section use pseudonyms.

## 3. Results

### 3.1. Meaningful Exposure

All parents reported daily TV (and other media) use, however many watched non-terrestrial channels rather than the three terrestrial channels that the advert aired on. Despite this, 13 of the 17 parents had seen and mentioned the TV advert when asked to recall anything about secondhand smoking that they had seen in the media in recent months, and two mothers who had seen the adverts quoted the campaign messages unprompted. One parent recalled hearing the radio advert, another had seen a campaign poster in the local pharmacy, but parents had not seen the community events or the website. Demonstrating their recall and understanding of the messages portrayed in the TV advert, participants summarised these in their own words:

“I think it’s quite good, it’s getting the message across. It’s not just smoking away from your kid, but it lingers as well, when you’re smoking round them anyway. It’s just as bad, smoking away from them. Because it’s still travelling round the house.”

*Cara*

“Just to take it away from your bairns (children). Don’t make them breathe your smoke. And, I agree with it. I do actually agree with it; don’t let your bairns breathe your smoke.”

*Louise*

Recall and comprehension of the messages about lingering smoke and child vulnerability were high both for parents who had seen the advert before and for the four parents who watched the advert for the first time in the interview.

### 3.2. Motivational Response

Most parents had explained that they attempted to protect their children from SHS by restricting smoking to one room and by opening a window in the pre-campaign interviews. Many, therefore, identified with the advert’s portrayal of a mother who smoked by her kitchen window to protect her child. For instance, Tania, who had recently quit smoking, described how the advert appealed to her because it “felt real” and reaffirmed her decision to quit smoking.

“I thought, that’s actually quite good...(…) “cause I thought, that’s actually more like it” cause it showed the actual child (…) I just remember it was more realistic (…) It was more about smoking within close proximity, and the effects it has when you think that closing the window is enough. (…) If I was still smoking, I’d have seen that and went, “oh god”, you know what I mean. You think it’s enough protection but it’s not.”

Mothers frequently referred to the boy’s lungs “*going black*” (Louise) in the advert, demonstrating its visual and emotional impact of the depiction of a situation close to their own. They appeared to identify with the mother and to see their own child(ren) in the boy.

“It’s definitely had an effect on me. I get guilty when I hear it, you know, (…), it makes it even more penetrable in your brain, you think it’s me.”

*Michelle*

Indeed, one mother who viewed the advert for the first time in the interview was reduced to tears and vowed never to smoke in her home again.

All were aware that the advert aimed to inform parents about the risk that their smoking in the home posed to their children’s health, responses to which varied from acceptance and guilt, to resistance. Parents accepted that the ideal strategy would be to only smoke outside, and reported feelings of guilt about their smoking in the home given the potential impact on their children’s health; guilt that watching the advert appeared to reinforce. Most mothers and one father talked about their emotional responses to the advert: “*it gets me every time*” as one mother said. For another mother, the guilt provoked by the advert was partly credited with reinforcing her wish to stop smoking:

“But the advert’s horrible. It makes you feel ten times worse. I don’t know seeing the kids lungs is just like that...because they’re starting to go...do they no go black? (…) That’s horrible. That’s definitely another reason to stop.”

*Keira*

For others, expressions of guilt were tempered by the statement that they were doing their best.

“It does make me feel guilty and bad, but I do my best to keep it away from her, so…”

*Louise*

Guilt was also tempered by references to the smoking practices of others perceived to be less responsible. For example, Tara described her friend’s smoking practices and her own attempts to intervene.

“Her youngest is a baby coming up for a year, and she smokes in front of her, sorry, from day one, she was in a Moses basket and she’s sitting in the living room having a cigarette and the baby’s in the Moses basket. And I’m like, what are you doing? You know, and I came up with my baby and she was like… I was like, are you wanting to kill your baby? (…) you have to be cruel to be kind, and then of course when my baby was up there (...) and she went to light up, and I was (makes a “no” noise). That’s the first time… like I say, I don’t tell people what to do in their own house, but I’m like… you might want to kill your baby, but you ain’t killing mine, and the smoke in this house is enough to kill the both of them.”

Commenting on the guilt the advert provoked, Michelle reasoned it was beneficial and justified as it might change parents’ smoking practices.

“I think it’s a good thing to guilt trip us into stopping, oh, definitely. I mean show us the horrors, you know, shouldn’t be doing it and especially with your kids. See those mothers that walk about with fags in their mouths pushing the prams, oh…”

Michelle includes herself in those who ‘should’ feel guilty, but then reverts to, and distinguishes herself from, the smoking mother with a pram as a signifier of an irresponsible mother. Admitting some, but resisting full, blame characterises such discourses. Others, like Sharon, claimed the advert had changed their attitudes to smoking in their home and its effect on their children.

“I think it’s more just like, you don’t know until you see things like that. You don’t, so your demeanour don’t change until you see something like that. Know what I mean, and then you do see it and it does kind of make you go, oh, I’ve been letting my bairn do that, I’ve been doing that with my bairn. So it’s not very nice, but I think that gets the point across very well. You understand that, it’s explaining enough to you, look just don’t smoke in your house at all, basically, it’s travelling.”

Three mothers claimed the campaign had altered where they smoked and/or increased their motivation to stop smoking. One of these mothers had misunderstood the smokefree home message and said she had started smoking in other rooms rather than the kitchen where she used to smoke:

“They say you don’t smoke in the same room as your kid...well, or in the kid’s bedroom but it does linger for five hours or so. So, I try to go in rooms that she doesn’t go in (now).”

*Keira*

In a clear display of resistance to the advert’s messages Monica and Lisa, both of whom had seen the advert before, crossed their arms and hardly looked at the screen when the advert played, shaking their heads. Refuting the messages in the campaign, Monica expressed her disbelief and disapproval:

“I don’t know. Like, they’re still saying, like, take it outdoors, but the smoke’s obviously still going to be on your clothes, so they’ve still got to… it doesn’t make any difference what you do really, eh. So it’s weird. (…) Oh, I know ‘breathe faster’, but if the smoke’s just lingering in there and it’s only a tiny wee bit, how can they be breathing in more, do you know what I mean, if you can’t see it or can’t smell it… like, if you can’t see it or can’t smell it, how do the people actually know it’s there, do you know what I mean. It’s weird. Just mad.”

Similarly, Lisa refuted the message that a child at some distance in another room or in a well-ventilated space like her own home would be at risk:

“And really I think that’s just pure scaremongering for mothers. I do know it affects them if they’re in the same room and stuff, but if you have like kind of ventilation in your house then I don’t believe that that actually happens. (…) I don’t think that’s right at all, that’s why I think it’s just scare mongering. Like my windows are open 24/7 a day every day of the week, like all my windows, the only window that gets shut is my daughter’s at night! But if they’re in the same area as you then obviously it’s going to affect them, or if they’re walking past you.”

### 3.3. Opportunity to Act

Smokefree homes were described as desirable, but impossible, for most parents given their current circumstances, which entailed supervising a very young and mobile child with little support or access to outside space; exposing them to other risks. Their current strategies to protect children from SHS were not seen as wholly effective but the best they could do. For example, Louise expressed her desire to smoke outside to protect her asthmatic daughter from SHS but as a single parent she felt her options were constrained:

“I do agree with it, but there are some people… Like, I’m a single mum with two kids and I can’t leave them in the house while I nip down the stairs for a fag, so hanging out the window is my only option. I think they’ve actually hit the nail on the head with the advert. But, the way I see it is, if they want people not to smoke, give everybody a house that’s got a front and back garden. That would be the only way for it to be possible for me to smoke and still look after my children would be to have a front and back door house.”

Mark, a single father, described the effect of the advert on his smoking behaviour as temporary because of his addiction to nicotine and limited accommodation:

“It made me think. It really did make me think. And I think it took a wee while for me to have another smoke in the house. But then it just goes out of your head, then you start smoking again. It’s the addiction. (…) I couldn’t go outside. I wouldn’t leave the house because of (son). If I had a garden, yeah, I’d stand at the back door. Close the door and have a cigarette. But there’s no way I can get outside. If I had a proper veranda, then I could go out there and close the door. But because there’s nowhere I can go to get outside I can’t smoke outside.”

In a similar vein, Michelle, another single mother, did not refute the advert’s message or the portrayal of how many parents smoke, but questioned the target of the campaign.

“It’s good. It’s all aimed I think at people that live in, you know, nice houses with to go outside and have a fag and the majority of people I know don’t live in a house with garden to go outside and have a nice cigarette. I live in a flat and say my neighbour on the sixth floor, she can’t go downstairs, she’s got to hang out her window as well. (…) The advert is very, kind of, pigeonholed at a certain type of smoker or type of property a smoker might have. Assumed that she’s got a partner and a house with a garden when the majority of single folk on low incomes are smokers, live in flats, they know this but they’ve got to, kind of, stretch it out and make it sound good.”

Furthermore, parents’ accounts were often resistant to the idea that they were solely responsible for their children’s SHS exposure, when they could spend significant amounts of time with other adult family members who smoked such as ex-partners and grandparents with less strict home smoking rules.

### 3.4. Alternative Solutions

Rather than going outside to smoke, parents sought other options to reduce their children’s SHS exposure. Most said they would like to quit: a wish supported by overlapping concerns about child health (SHS exposure and role-modelling smoking), their own health, and family finances, which were significantly stretched for all. Quitting was perceived to be a great challenge.

“(Quitting)’s not that easy to do either. I know people that have smoked for years and years, even me I can’t just wake up one day and go I’m not going to smoke, because that is not going to work. And I’ve tried to stop a few times and it just doesn’t work.”

*Lisa*

Further, the exponential rise in the UK in e-cigarette use during the study period was, to some extent, reflected in parents’ accounts with some using or planning to start using e-cigarettes instead of cigarettes. For example, Sharon described using e-cigarettes inside her home to protect her son who had recently been diagnosed with asthma, while smoking cigarettes when outside her home.

“Well, with the normal fags, obviously that… (son) can inhale the smoke, or whatever, and the fumes and stuff. But with the electric one, he can’t.”

Events other than the mass media campaign had also increased some parents’ awareness of SHS and changed their smoking practices between the two interviews. The rapidly changing lives described by many parents in the first interview [30] which included frequent house moves, relationship breakdowns, and subsequent increased social isolation, had continued for some. Since the first interview, two participants had moved again, one mother had moved in with her partner, two previously single mothers had new partners, and two mothers were pregnant. One of the children had been diagnosed with asthma and another with leukaemia. All these changes, particularly the latter, were reported to have led to reductions or increases in smoking, and other changes in parents’ smoking practices.

## 4. Discussion

The findings of this study add to our understanding of how disadvantaged parents respond to mass media campaigns on SHS. The study has several limitations. It involved a small number of purposively recruited parents from five communities in Scotland with a diverse socioeconomic profile who may not be generalizable to the wider population of parents who smoke. While the parents were not shown the media advert in the first interview or told about the imminent media campaign they may have been more receptive to the advert after participation in the first interview about smoking in the home. Additionally, Scotland has had comprehensive smokefree public places’ legislation since 2006, so the findings may not be generalizable to countries with less comprehensive, or more recent, smokefree legislation. Despite these limitations, the findings show that disadvantaged parents engaged with the visual, emotive, and real-life circumstances of parents attempting to protect their children from SHS in the ‘Right Outside’ campaign. However, despite accepting the campaign’s messages and a reported high motivation to protect their children from SHS exposure, parents’ opportunities to act on the messages were constrained by their domestic circumstances. Unlike the mother in the advert who had a partner (and possibly a garden), many parents in the study did not have partners, gardens, or safe outdoor spaces and, therefore, could not leave their children unattended to smoke outside. Previous studies have found that disadvantaged smoker’s responses to mass media campaigns can differ with more limited understandings and recall of messages, less motivation, and more limited opportunities to act [20]. This study indicates that a TV advertisement, when designed to reflect the practices of disadvantaged parents who smoke in the home but want to protect their children from SHS, can constructively engage parents, but their limited opportunities to act on the messages remains problematic.

Rather than smoking outside, most parents sought and discussed alternative ways of protecting their children, including quitting, which many thought they would find difficult. Parents in similar disadvantaged circumstances may, therefore, need to be offered alternative options which health and social care professionals need to be committed to and confident in providing. Regardless of parents’ circumstances or gender, health and social care professionals should provide parents with SHS advice and cessation support. Parents’ attempts to quit, reduce and/or protect their children from their smoking within these challenging circumstances should be supported and not undermined [29]. Consideration should, therefore, be given to offering and supporting potential harm reduction options, such as temporary use of e-cigarettes and nicotine replacement therapy (NRT), for parents who feel unable to quit and cannot smoke safely outside, to create smokefree homes. Avoiding health risks has become a moral obligation for parents, particularly mothers [32]. The findings suggest that tobacco control’s attempts to de-normalise smoking may have become embedded in these parents’ view of themselves and other parents. We, therefore, propose that the predominant focus on parents (particularly mothers) who smoke in SHS mass media campaigns [18], should be expanded to encompass fathers and grandparents who smoke indoors, whom mothers can often feel powerless to affect [29]. This would also be likely to increase campaign effectiveness and avoid the further stigmatisation of disadvantaged parents who smoke [29].

## 5. Conclusions

Mass media campaigns like “Right Outside”, with visual, emotive real-life circumstances portraying parents attempting to act responsibly to reduce children’s exposure to SHS, may be effective in engaging disadvantaged parents with SHS messages. However, the impact on parents smoking practices may be limited given their challenging home environments and circumstances. Alternative options and additional support for parents in disadvantaged circumstances are, therefore, needed to avoid undermining their wishes and actions to protect their children.

## Supplementary Materials

The following are available online at www.mdpi.com/1660-4601/13/9/901/s1, Protecting Young Children in Disadvantaged Households from Secondhand Smoke: Identifying Barriers and Levers to Smokefree Homes.
