# Supplementary material for: Disadvantaged Parents’ Engagement with a National Secondhand Smoke in the Home Mass Media Campaign: A Qualitative Study
Source: Int J Environ Res Public Health. 2016 Sep 9;13(9):901. doi: 10.3390/ijerph13090901 (PMC5036734; doi:10.3390/ijerph13090901)
Supplement: Supplementary file 1 [file ijerph-13-00901-s001.pdf]

Supplementary Materials: Disadvantaged Parents' Engagement with a National Secondhand Smoke in the Home Mass Media Campaign: A Qualitative Study

Neneh Rowa-Dewar and Amanda Amos

Protecting Young Children in Disadvantaged Households from Secondhand Smoke: Identifying Barriers and Levers to Smokefree Homes

Phase 2

Follow-up Interview Topic Guide

Interviews will be semi-structured; the order and phrasing of the questions will depend on the participant's responses.

Introduction

- Last time we met we spoke about your experiences of smoking in the home (and the car) before and after you had (name of child) and any changes to where and when you and others smoke. I would like to ask you about any changes to your circumstances and smoking restrictions and about anything you might have heard or seen in the media about smoking in the home since then.
- We will talk for about an hour.
- You can withdraw at any time from the interview as a whole or you can choose not to answer certain questions.
- I would like to record the conversation to write up and look at later. Only the research team will ever hear the tape and your name and address will not be on the transcript.
- I would like to use some of your words in my final report and any presentations and articles I write for academic journals but will anonymise them.
- Obtain informed consent.

1. Update

Thanks for letting me interview you again. Can you tell me a bit about what has happened since we last spoke? Last time we spoke you (smoking status, living arrangements and smoking restrictions). Do you still smoke/have you started again? Do you still live in the same place and with (name of partner and children)?

2. Smoking in the Home and Car

Have your smoking restrictions changed in any way? Why? (If yes, ask participant to draw another floor plan and show where and when they currently smoke).

3. Awareness and Extent of SHS Effects

Do you feel smoking affect others close to those who smoke? (Probe re: age of person, child/adult, health, in what circumstances etc.). Where have you heard that (media, family and friends, practitioners)?

Probe re: specific ad messages—why are children more sensitive (kids breathe faster) and smoke dispersal (smoke reaches everywhere).

4. Recall and Response to Media SHS Message(s)

Recall

What have you seen or heard in the media about smoking in the home or car? TV? Radio? Newspapers and magazines? Billboards?

What was the message?

Response and Application

What did you think of the message? Could/have you applied that in your life? If so, was it/would it be challenging? Would you like to if possible? Why/why not? How about your family (have they seen it, talked about it, responded to it)?

Prompt for SG Campaign

Showing visual of the final frame: You can choose whether your child breathes secondhand smoke or clean air. For your kids' sake take it right outside.

What is your response to this message?

Play TV advert.

What do you think about this? How do you think it could be improved?

8. Practitioner Response/Support

Has anyone (else) asked about/given advice about your smoking since you were pregnant? Was that helpful/unhelpful?

9. Anything Else You Would Like to Add? Or Ask Me about?

© 2016 by the authors; licensee MDPI, Basel, Switzerland. This article is an open access article distributed under the terms and conditions of the Creative Commons by Attribution (CC-BY) license (http://creativecommons.org/licenses/by/4.0/).
